# Antidote for Carbolic Acid

**Published:** 1892-09

**Authors:** 


ANTIDOTE FOR CARBOLIC ACID.—It is now reported that an efficient antidote for poisoning by carbolic acid has been discovered by an Italian tailor, who swallowed by mistake thirty grammes of carbolic acid. Dr. Morett, of Ancona, using a rubber sound, immediately introduced by slow degrees into the patient's stomach a strong solution of sulphate of soda, which forms with carbolic acid a harmless mixture. In an hour's time the patient, who had been in a most critical condition, began to revive, the pulse became better and the respiration improved. Inhalations of ammonia were then used to hasten up the process, and little by little the poisoned man rallied so much that an emetic, followed by a dose of lime water, finished the cure.—*Ex.*

